# The Impact of Visitation Guidelines During COVID-19 on Well-Being and Daily Life in Nursing Homes

**DOI:** 10.1093/geroni/igab046.1575

**Published:** 2021-12-17

**Authors:** Debby Gerritsen, Ramona Backhaus, Bram de Boer, Judith Urlings, Raymond Koopmans, Jan Hamers, Hilde Verbeek

**Affiliations:** 1 Radboud University Medical Centre, Nijmegen, Gelderland, Netherlands; 2 Maastricht University, Maastricht, Limburg, Netherlands; 3 Living Lab in Ageing and Long-Term Care, Maastricht, Limburg, Netherlands

## Abstract

Nursing homes across the world have taken very restrictive measures, including a ban for visitors, to prevent and control COVID-19 infections. This study reports on findings of a study investigating guidelines on allowing visitors in nursing homes and the impact on residents’ well-being, family caregivers and staff. In total, 76 nursing homes in the Netherlands were followed using a survey study, including three waves of data collection in (May 2020, September 2020, March 2021. Results indicated a negative impact of a visitation ban for residents’ overall well-being. There was a variety in guidelines of allowing visitors in nursing homes, and showed that safe visiting was possible during the COVID-19 pandemic. Staff perceived a fragile balance between infection prevention and the impact of restriction on residents. In conclusion, a general ban for visitors is not necessary and may do more harm than good for residents living in nursing homes.

